# Nobiletin downregulates the SKP2-p21/p27-CDK2 axis to inhibit tumor progression and shows synergistic effects with palbociclib on renal cell carcinoma

**DOI:** 10.20892/j.issn.2095-3941.2020.0186

**Published:** 2021-02-15

**Authors:** Tingting Chen, Liu Liu, Yonghong Zou, Xiaoyan Hu, Wenfeng Zhang, Tao Zhou, Xi Luo, Weihua Fu, Jie Xu

**Affiliations:** 1 Department of Urology, Xinqiao Hospital of Army Medical University, Chongqing 400037, China; 2 Department of Nephrology, Tongji Hospital, Tongji Medical College, Huazhong University of Science and Technology, Wuhan 430040, China; 3 Department of Reproductive Medicine, Ji’an Central People’s Hospital, Ji’an 343100, China; 4 Department of Infectious Disease, the First Affiliated Hospital, Nanchang University, Nanchang 330001, China; 5 Department of Oncology, Southwest Hospital of Army Medical University, Chongqing 400038, China

**Keywords:** Nobiletin, SKP2, palbociclib, synergistic, renal cell carcinoma

## Abstract

**Objective:**

Natural extracts, including nobiletin, have been reported to enhance the efficacy and sensitivity of chemotherapeutic drugs. However, whether and how nobiletin affects tumor growth and progression in renal cell carcinoma (RCC) are still unclear.

**Methods:**

Cell proliferation, cell cycle and apoptosis analyses, colony-formation assays, immunoblotting analysis, and qRT-PCR analysis were performed to investigate how nobiletin affected RCC cell proliferation *in vitro*. The nude mouse model was used to test the efficacy of nobiletin alone or in combination with palbociclib.

**Results:**

Nobiletin inhibited cell proliferation by inducing G1 cell cycle arrest and cell apoptosis in RCC cells. Mechanistically, nobiletin decreased SKP2 protein expression by reducing its transcriptional level. The downregulated SKP2 caused accumulation of its substrates, p27 and p21, which further inhibited the activity of the G1 phase-related protein, CDK2, leading to inhibition of cell proliferation and tumor formation. A higher SKP2 protein level indicated less sensitivity to the CDK4/6 inhibitor, palbociclib. A combination of nobiletin and palbociclib showed a synergistic tumor inhibition *in vitro* and in an *in vivo* model.

**Conclusions:**

Nobiletin downregulated the SKP2-p21/p27-CDK2 axis to inhibit tumor progression and showed synergistic tumor inhibition effects with the CDK4/6 inhibitor, palbociclib, on RCC, which indicates a potential new therapeutic strategy.

## Introduction

Renal cell carcinoma (RCC) is the 6th most common cancer in men and the 10th in women, accounting for 5% and 3% of all new cancer cases, respectively^1^. Despite a higher proportion of indolent and localized tumors identified partly due to widespread use of abdominal imaging techniques, there are still up to 17% of patients with distant metastases at the time of diagnosis^2^. In addition, approximately 25% of patients with localized RCC develop distant metastasis after radical surgery. Once progressed to metastatic RCC (mRCC), the treatment is still an intractable problem, despite the introduction of novel agents targeting different pathways including angiogenesis, mTOR inhibitors, and immune checkpoint inhibitors. Compared with cytokine treatment (interferon alpha or interleukin-2), targeted therapies have significantly improved the clinical outcomes of mRCC^3^. Sunitinib, as a first-line therapy for RCC, achieved significantly longer progression-free survival (11 *vs.* 5 months), overall survival (OS) (26.4 *vs.* 21.8 months), and a better response percentage (31% *vs.* 6%) than the interferon alpha group^4,5^. However, due to the intratumor and intertumor heterogeneities of RCC, the objective response percentages of current first-line agents are approximately 30% or lower^3,6^. Moreover, almost all patients eventually suffer drug resistance, which suggests that new targets and treatment strategies need to be identified to effectively treat these patients.

Many natural extracts are potential antitumor drugs because of their low toxicity and few side effects^7,8^. Some extracts have anti-tumor pharmacological effects and can be used as a regulator of multi-drug resistance, to increase the sensitivity of cancer cells to chemotherapy^9^. Nobiletin is a typical example, which is an O-methylated flavonoid found mainly in citrus peel. Previous studies have shown that nobiletin significantly inhibited tumor cell growth and metastasis both *in vitro* and *in vivo* through multiple pathways^10,11^. Nobiletin can also significantly inhibit cell proliferation by attenuating the expression of cyclin D1, CKD2, CKD4, and E2F^12^. Nobiletin inhibits RCC cell viability and hypoxia-induced migration, which might be induced by the SRC/AKT, NF-κB, and Wnt/β-catenin signaling pathways^13,14^. Although nobiletin has been reported to induce G0/G1 cell cycle arrest in RCC cells, the mechanism of action is still unclear^14^.

Cell-cycle dysregulation is prevalent in multiple malignancies, including RCC^15^. G1/S cell cycle transition is mainly regulated by two cyclin/CDK complexes, cyclinD-CDK4/6 and cyclinE-CDK2, which have been reported to trigger G1/S transition by promoting RB (retinoblastoma) phosphorylation^16^. Abnormal CCND-CDK4/6-INK4-RB signaling has been involved in tumorigenesis and in tumors^17–21^, and represents a valid therapeutic target^16^. Palbociclib is an orally active, potent, and selective inhibitor of CDK4 and CDK6, which blocks RB phosphorylation at low drug concentrations^22^. Palbociclib can inhibit RCC cell proliferation at nanomolar concentrations^23^, and has been tried in phase II studies to treat various types of solid tumors^24–26^. However, the effects of CDK4/6 inhibitors have been limited by the other bypass signal^27^. Some studies have shown that palbociclib blocked cells in the G1 phase by inhibiting CDK4/6 activity, but it did not inhibit CDK2 activity, and the inhibitory effect of RB phosphorylation could be reversed by CDK2 to produce drug resistance^28^, which indicated that a combination CDK2 inhibitor with palbociclib may increase the therapeutic effect.

In this study, we found that nobiletin induced G1-phase cell cycle arrest, apoptosis, and proliferative inhibition in RCC cells. Nobiletin downregulated mRNA expression of SKP2 by upregulating the transcription factor, FOXO3A, leading to accumulation of p21 and p27 and the reduction of CDK2, to inhibit cell proliferation and tumor formation. We further found that the sensitivity of the CDK4/6 inhibitor, palbociclib, was negatively associated with SKP2 protein levels. Furthermore, a combination of nobiletin and palbociclib showed synergistic lethality, which suggests this combined regimen may be a therapeutic strategy for RCC.

## Materials and methods

### Reagents and cell cultures

Nobiletin, palbociclib, MG-132, and cycloheximide were purchased from Selleck Chemicals (Houston, TX, USA), and dissolved in dimethyl sulfoxide (DMSO) (Amresco, Solon, OH, USA) and stored at -20 °C. The RCC lines (786-O, 769-P, OSRC-2, and Caki-1) and the immortalized epithelial renal cell line (HK-2) were purchased from the Zhong Qiao Xin Zhou Biotechnology (Shanghai, China). The 786-O, 769-P, and OSRC-2 cell lines were routinely maintained in RPMI-1640 medium. The Caki-1 cell line was cultured in McCoy’s 5A medium, and the HK-2 cell line was cultured in KFSM medium. All media were supplemented with 10% fetal bovine serum and 1% penicillin-streptomycin except KFSM medium. All media were purchased from Gibco (Gaithersburg, MD, USA). The cells were cultured at 37 °C in a humidified atmosphere containing 5% CO_2_. All cell lines were tested and were shown to be free of mycoplasma contamination.

### Immunoblotting (IB) and antibodies

For direct IB analysis, the cells were lysed in radioimmunoprecipitation assay (RIPA) buffer with phosphatase inhibitors. The following primary antibodies were used against: cleaved caspase-3 (Asp175) (5A1E) (#9664), cleaved caspase-8 (Asp391) (18C8) (#9496), RB (#9309), p-RB (Ser870/811) (#8516), CDK2 (#2546), p-CDK2 (#2561), Bcl-2 (#15071), Bax (#5023), p21 (#2947), p27 (#3686), cyclin D1 (#55506), cyclin E (#4136), p44/42 MAPK (Erk1/2) (137F5) (#4695), phospho-p44/42 MAPK (Erk1/2) (Thr202/Tyr204) (D13.14.4E) XP (#4370), Akt (pan) (40D4) (#2920), phospho-Akt (Ser473) (D9E) XP (#4060), PI3 kinase p110α (C73F8) (#4249), cyclin A2 (#91500), and cyclin B1 ( #4135), all purchased from Cell Signaling Technology (Danvers, MA, USA). Antibodies to CDK4 (#11026-1-AP), CDK6 (#14052-1-AP), SKP2 (#15010-1-AP), p53 (1C12) (#2524), P16-INK4A (#10883-1-AP), EGFR (#18986-1-AP), and glyceraldehyde 3-phosphate dehydrogenase (GAPDH) (#60004-1-Ig) were purchased from Proteintech (Rosemont, IL, USA). The anti-FLAG was obtained from Sigma-Aldrich (St. Louis, MO, USA). All secondary antibodies were purchased from Cell Signaling Technology, Danvers, MA, USA.

### Plate colony-forming assay

Cells were seeded into 6-well plates (Corning, Corning, NY, USA) at a density of 500 cells/well. After 24 h, cells were treated with or without nobiletin for 48 h. The nobiletin-containing medium was then removed and replaced by complete medium, followed by incubation at 37 °C for 10–14 days. The colonies were fixed with 4% paraformaldehyde, stained with Crystal Violet, and counted. The colonies composed of ≥ 50 cells were counted using a microscope. Each experiment was conducted in triplicate.

### Lentivirus or transient transfection

For transient transfection, the cells were seeded in an antibiotic-free medium at 37 °C for 24 h and transfected with constitutively active SKP2 plasmid or empty vector DNA using Lipofectamine-2000 transfection reagent (Life Technologies, Carlsbad, CA, USA) according to the manufacturer’s instructions, and treated 48 h after transfection. The lentivirus used for SKP2 silencing was purchased from Genepharma (Shanghai, China). The cells were transfected with lentivirus according to the manufacturer’s protocol.

### Apoptosis analysis

An annexin V assay was performed according to the manufacturer’s instructions (Life Technologies, Carlsbad, CA, USA). Nobiletin- and/or palbociclib-treated cells were collected, and used for annexin V-fluorescein isothiocyanate (FITC)/propidium iodide (PI) staining. The samples were then analyzed using flow cytometry within 1 h. Each experiment was conducted in triplicate.

### Cell cycle analysis

Cells were collected at the indicated time points after nobiletin and/or palbociclib treatment and fixed with ice-cold 70% ethanol, then stored at 4 °C overnight. After washing twice with phosphate-buffered saline, FxCycle PI/RNAse staining solution (Invitrogen, Carlsbad, CA, USA) was used for detection of the cell cycle according to the manufacturer’s protocol. The stained cells were subjected to cell cycle analysis using a flow cytometer and analyzed for cell cycle phases with the C6 Accuri software system (BD Biosciences, San Jose, CA, USA). Each experiment was conducted in triplicate.

### Cell viability and cell proliferation assays

The Cell Counting Kit-8 (CCK-8) assay (Dojindo Molecular Technologies, Kumamoto, Japan) was used to assess the cell viability. The cell concentration was adjusted to 2 × 10^3^ cells/well, and the cells were seeded into 96-well plates, followed by 24 h of culture at 37 °C in an atmosphere with 5% CO_2_, then were treated with various concentrations of palbociclib or nobiletin and maintained in culture for 48 h. After removing the culture medium, the CCK-8 reaction solution was added according to the manufacturer’s instructions, and the absorbance was measured at 450 nm. The half-maximal inhibitory concentration (IC_50_) value is a critical index of the dose-response curve. Prism statistical software (GraphPad, San Diego, CA, USA) was used to calculate the IC_50_ values and to plot dose-response curves. According to IC_50_ values, the cells was treated with the given concentration of nobiletin, and cultivated for a further 0, 1, 2, 3, and 4 days. At different times after cell plating, the cells were subjected to the CCK-8 assay, according to the manufacturer’s instructions. Each experiment was conducted in triplicate.

### Cycloheximide chase analysis

Cycloheximide chase analysis was performed as described previously to define the effect of nobiletin on the stability of the SKP2 protein^29^. Briefly, 786-O and 769-P cell lines (5 × 10^5^ cells) were exposed to 100 μM and 25 μM nobiletin for 24 h, respectively, and followed by 24 h treatment with cycloheximide (50 μg/mL) (MCE, #HY-12320, Monmouth Junction, NJ 08852, USA) to stop de novo protein synthesis. SKP2 levels at 0, 4, 8, 12, and 24 h following cycloheximide co-treatment were then determined using IB analysis. Each experiment was conducted in triplicate.

### Quantitative real-time PCR (RT-PCR)

Total RNA was isolated from control and nobiletin-treated cells using RNAisoPlus (Takara, Otsu, Japan), according to the manufacturer’s instructions. Reverse transcription of the extracted RNA to corresponding complementary DNA was performed using a PrimeScript RT reagent Kit with gDNA Eraser (Takara). The qRT-PCR was performed with a QuantiNova™ SYBR® Green PCR Kit (Qiagen, Hilden, Germany) on an Applied Biosystems 7900HT Real-Time PCR System (Applied Biosystems, Foster City, CA, USA). The housekeeping gene, *GAPDH*, was used as a loading control. The following forward and reverse primers were used: SKP2 forward-CAGGCCTAAGCTAAATCGAGAG, SKP2 reverse-CTGGCAATGGT GGTGAAATG; GAPDH forward-AG CCTTCTCCATGGTTGGTGAAGAC, GAPDH reverse-CGGAGTCAACGGATTTG GTCGTAT. Each experiment was conducted in triplicate.

### Immunohistochemistry (IHC) staining

IHC staining of mice tumors was performed as described previously^30^. Briefly, after deparaffinization, rehydration, antigen retrieval, and blocking, the tissue slides were incubated overnight at 4 °C with the indicated antibodies. The following primary antibodies were used: anti-Ki-67 (Cell Signaling Technology, #9449), anti-p27, and anti-cleaved-capase-3.

### *In vivo* xenograft model

Four- to six-week-old BALB/c athymic nude mice (nu/nu, female) were used, with each experimental group consisting of 5 mice. All animal experiments were conducted according to a protocol approved by the university committee for use and care of animals. A total of 2 × 10^6^ 786-O cells were mixed 1:1 with Matrigel (BD Biosciences) in a total volume of 200 μL, and were injected subcutaneously into the right flank side of nude mice. Nude mice were treated with vehicle, nobiletin (40 mg/kg/day, every day, per gavage), palbociclib (120 mg/kg/day, every day, per gavage), and nobiletin + palbociclib when the tumor size reached about 100 mm^3^. Nude mice were euthanized after 21 days of treatment. The growth of tumor was measured twice a week and the average tumor volume (TV) was calculated according to the equation: TV = (L × W^2^)/2. The protocol was approved by the Laboratory Animal Welfare and Ethics Committee of the Third Military Medical University of China (Approval No. AMUWCE2019417).

### Statistical analysis

All data are shown as the average ± standard deviation (mean ± SD) and each experiment was independently repeated at least 3 times. All statistical analyses were performed using the GraphPad Prism, version 5.0. The statistical significance of differences between groups was examined by one-way analysis of variance followed by Tukey’s multiple comparison procedure or Student’s *t*-test. *P* < 0.05 was considered to be statistically significant. Both CalcuSyn software^31^ and Jin’s formula^32^ were used to evaluate the synergistic effects of drug combinations. Jin’s formula is Q = Ea + b / (Ea + Eb - Ea × Eb), where Ea + b represented the cell proliferation inhibition rate of the combined drugs, while Ea and Eb represented the rates for each drug, respectively. A value of Q = 0.85–1.15 indicated a simple additive effect, while Q > 1.15 indicated synergism. Combination index (CI) plots were generated using CalcuSyn software (http://www.biosoft.com/w/calcusyn.htm).

## Results

### Nobiletin inhibits cell proliferation of RCC cells

**Figure 1:**
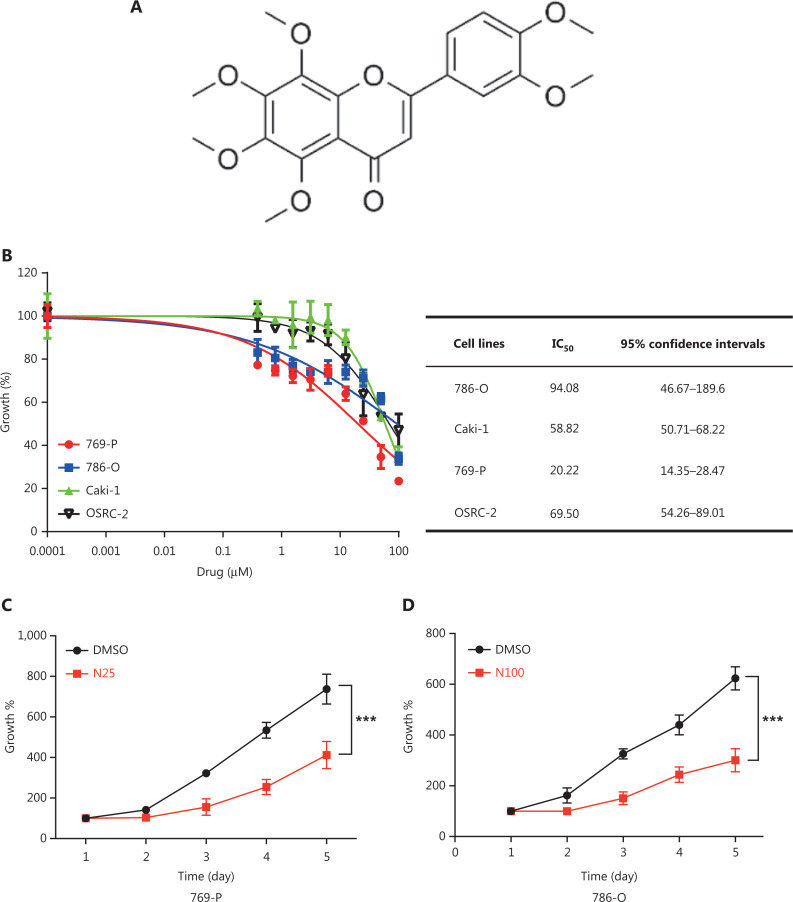
Anti-proliferative action of nobiletin on renal cell carcinoma (RCC) cell lines. (A) The structure of nobiletin. (B) Dose-response curves of nobiletin against RCC cell lines, after treatment for 48 h. The cell viability was detected with the Cell Counting Kit-8 (CCK-8) assay. (C–D) Nobiletin inhibited RCC cell proliferation in a time-dependent manner. Cultured 769-P and 786-O cell lines were treated with 25 μM and 100 μM nobiletin for different times, respectively, and then their cellular viabilities were determined using the CCK-8 assay. Data are presented as the means ± SD. ***P < 0.001, compared with the control group; *n* = 3.

The chemical structure of nobiletin is shown in **Figure 1A**. We first performed a CCK-8 assay to identify the effects of nobiletin in RCC and found that nobiletin significantly inhibited cell growth of RCC cell lines in a dose-dependent manner (**Figure 1B**). The results showed that the IC_50_ response of nobiletin was lower in the 769-P cell line (IC_50_ = 20.22 μM) and higher in the 786-O cell line (IC_50_ = 90.48 μM) (**Figure 1B**). We also found that nobiletin inhibited cell proliferation of 786-O and 769-P cell lines in a time-dependent manner (**Figure 1C and 1D**, *P* < 0.001).

### Nobiletin induces G1-phase arrest, cell apoptosis, and inhibits colony-formation in RCC cells

**Figure 2:**
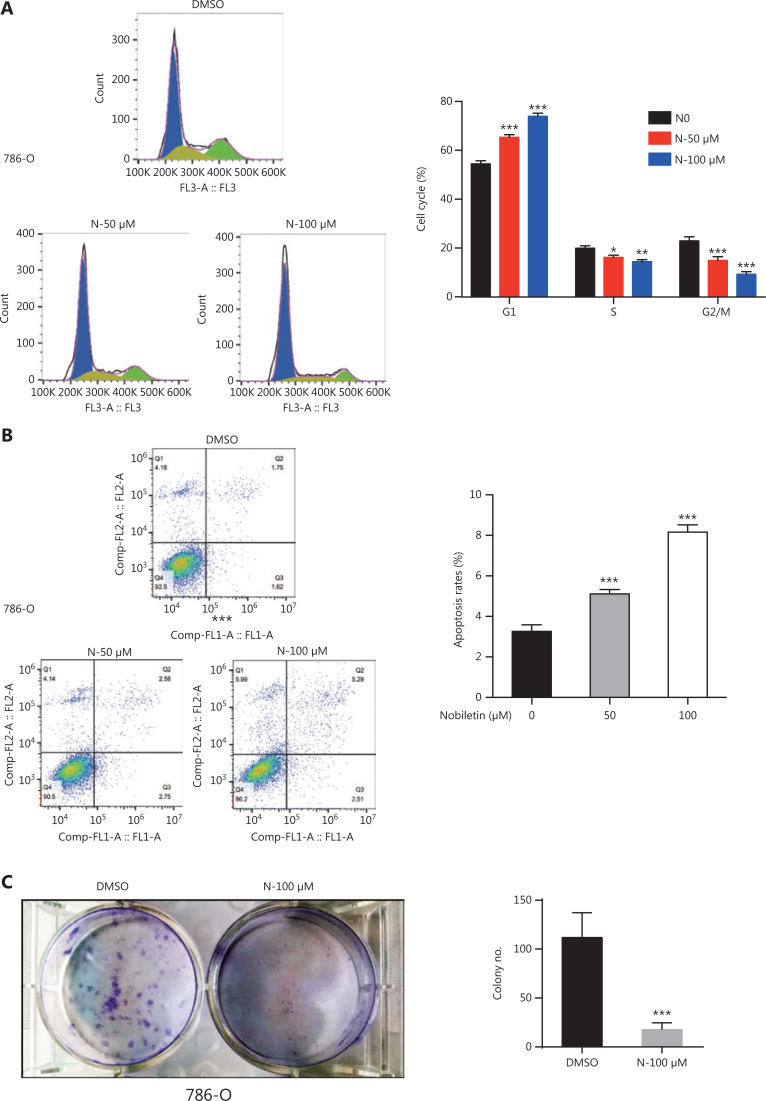
Nobiletin induced G1-phase cell cycle arrest, apoptosis, and colony-forming inhibition in the 786-O cell line. (A) Nobiletin arrested cell cycle progression at the G1-phase cell cycle in the 786-O cell line. The 786-O cell line was treated with 50 or 100 μM nobiletin for 48 h, and cell cycle distributions were then analyzed by flow cytometry. (B) Nobiletin induced apoptosis in the 786-O cell line. Apoptosis in the 786-O cell line was examined after 48 h of treatment with 50 or 100 μM nobiletin, by annexin V- fluorescein isothiocyanate/propidium iodide binding, and analyzed by flow cytometry. (C) Nobiletin significantly suppressed colony formation in the 786-O cell line. The 786-O cell line treated with 100 μM nobiletin for 48 h was allowed to proliferate in drug-free culture medium for 10˜14 days to form colonies, followed by Crystal Violet staining for scoring colonies. Quantitative results were obtained from the number of colonies. Data are presented as the means ± SD. *P < 0.05; **P < 0.01; ***P < 0.001, compared with the control group; *n* = 3.

We next determined whether nobiletin affected cell cycle distribution or apoptosis in RCC cells. The flow cytometry assays showed that nobiletin induced accumulation of G1-phase cells (**Figure 2A and Supplementary Figure S1A**) and promoted cell apoptosis in a dose-dependent manner in 786-O and 769-P cell lines (**Figure 2B and Supplementary Figure S1B**, *P* < 0.001). In addition, nobiletin significantly inhibited the colony formation ability of 786-O and 769-P cell lines (**Figure 2C and Supplementary Figure S1C**, *P* < 0.001). Nobiletin may have inhibited cell proliferation by inducing cell cycle arrest and apoptosis.

### Nobiletin regulates G1 phage proteins and increases apoptosis-related proteins

**Figure 3:**
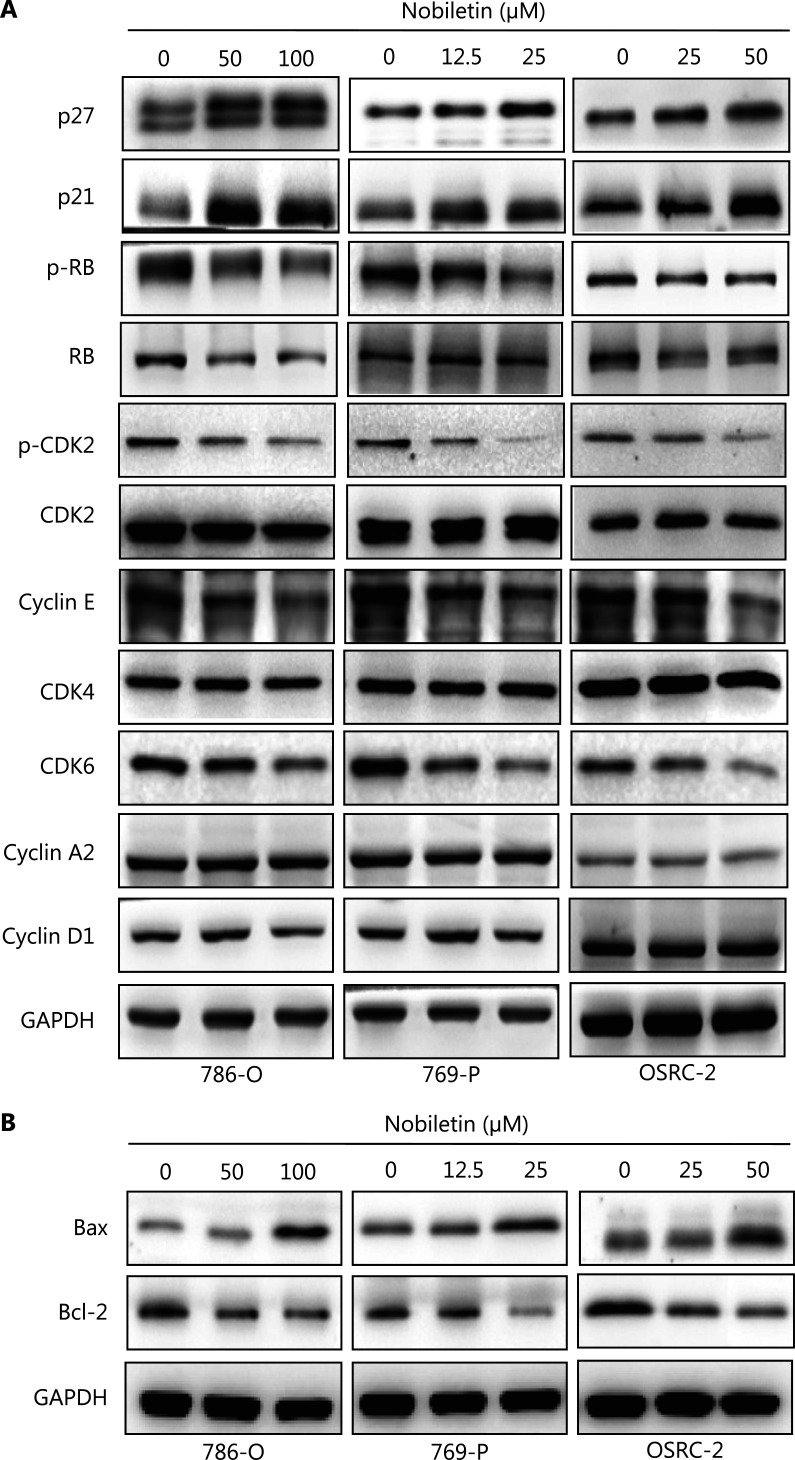
The effects of nobiletin on the expression of cell cycle regulatory protein and apoptosis-related protein in renal carcinoma cells (RCCs). (A) The effect of nobiletin on the cell cycle regulatory protein expression in RCC cell lines. RCC cell lines were treated with different doses of nobiletin (50 and 100 μM for the 786-O cell line, 12.5 and 25 μM for the 769-P cell line, and 25 and 50 μM for OSRC-2 cell line) for 48 h, followed by determination of related-protein expression using IB. (B) The effect of nobiletin on expression of apoptosis-related protein expression in RCC cell lines. RCC cell lines were treated with different doses of nobiletin (50 and 100 μM for the 786-O cell line; 12.5 and 25 μM for the 769-P cell line; and 25 and 50 μM for the OSRC-2 cell line) for 48 h, followed by determination of related protein expression using IB analysis. Data are presented as the means ± SD. Glyceraldehyde 3-phosphate dehydrogenase levels served as the control for equal protein loading.

To clarify the mechanism of nobiletin involved in cell cycle arrest and apoptosis, we characterized the expression of G1 phase and apoptosis-related proteins upon nobiletin treatment. Consistent with G1-phase arrest, G1-phase checkpoint proteins p21 and p27 were increased by nobiletin treatment (**Figure 3A and Supplementary Figure S2A**). Nobiletin had no effect on CDK2, CDK4, and cyclin D1 levels, but reduced the expressions of p-CDK2, RB, and p-RB levels with increasing doses, in RCC cell lines (**Figure 3A and Supplementary Figure S2A**), indicating that G1/S transition was blocked. Nobiletin also attenuated the expression of cyclin E and p-RB (**Figure 3A and Supplementary Figure S2A**). Our findings suggested that nobiletin induced cell cycle arrest during the G1 phase by increasing P21/p27, but inhibited CDK2 and RB activities. In addition, the decrease in Bcl-2 levels and the increase in Bax levels (**Figure 3B and Supplementary Figure S2B**) confirmed the apoptotic effect of nobiletin on RCC cell lines.

### SKP2 downregulation is fundamental for nobiletin-induced anti-proliferation in RCC cells

**Figure 4:**
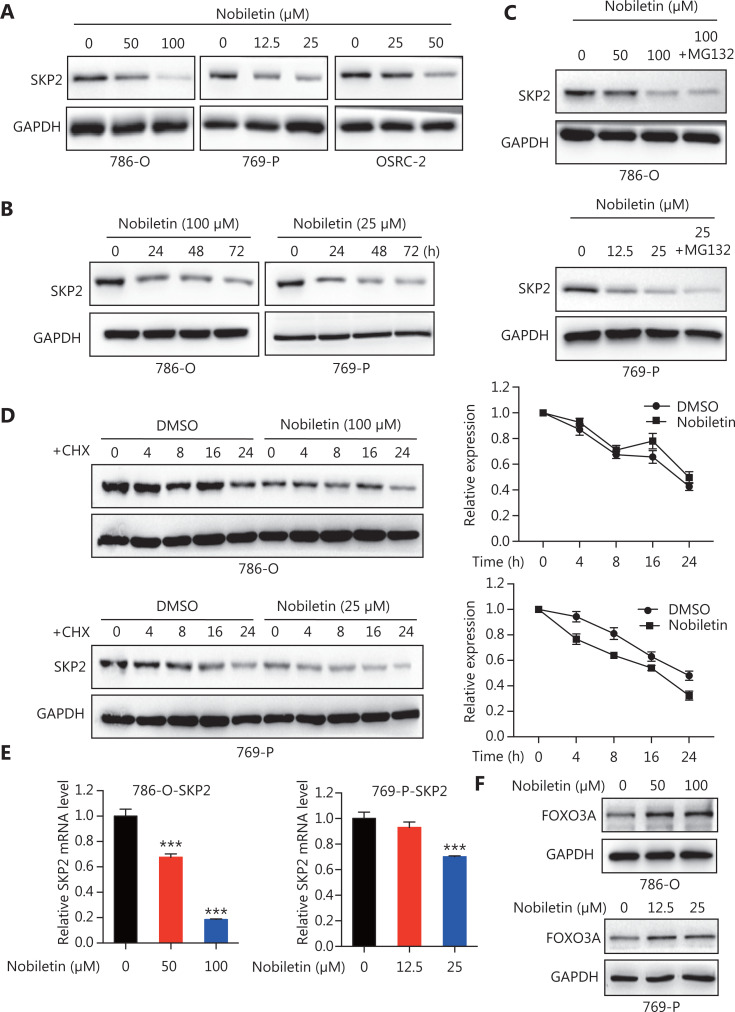
Nobiletin inhibited cell proliferation through downregulation of SKP2 in renal carcinoma cell (RCC) cell lines, and SKP2 degradation was mediated by mRNA degradation. (A) Nobiletin dose-dependently downregulated SKP2 levels in RCC cell lines. RCC cell lines were treated with different doses of nobiletin (50 and 100 μM for the 786-O cell line; 12.5 and 25 μM for the 769-P cell line; and 25 and 50 μM for the OSRC-2 cell line) for 48 h, followed by determination of related protein expression using IB analysis, followed by SKP2 IB. (B) Nobiletin time-dependently downregulated SKP2 levels in RCC cell lines. Cultured 769-P and 786-O cell lines were treated with 25 μM and 100 μM nobiletin, respectively, for different times, and followed by SKP2 IB. (C) Blockade of proteasome-mediated degradation failed to restore SKP2 downregulation by nobiletin. RCC cell lines were treated with different doses of nobiletin (50 and 100 μM for the 786-O cell line, and 12.5 and 25 μM for the 769-P cell line) for 24 h. The proteasome inhibitor, MG-132, was then added to nobiletin-treated cells and MG-132 co-treatment was allowed to occur for 2 h, followed by SKP2 IB. (D) Nobiletin did not destabilize SKP2 levels in RCC cell lines. The 786-O and 769-P cell lines were treated with 100 μM and 25 μM nobiletin for 24 h, respectively, and then subjected to cycloheximide [dose?] chase analysis. The levels of SKP2 at 0, 4, 8, 16, and 24 h after cycloheximide treatment were determined using IB analysis. (E) Nobiletin downregulated SKP2 levels by regulating SKP2 mRNA levels in RCC cell lines. The 786-O and 769-P cell lines were treated with 100 μM and 25 μM nobiletin for 48 h, respectively, and followed by determination of SKP2 mRNA levels using qRT-PCR analysis. (F) FOXO3, as a transcription factor, was upregulated by nobiletin. RCC cell lines were treated with different doses of nobiletin (50 and 100 μM for the 786-O cell line, and 12.5 and 25 μM for the 769-P cell line) for 48 h, followed by FOXO3A IB. Data are presented as the means ± SD. ***P < 0.001, compared with the control group; *n* = 3. Glyceraldehyde 3-phosphate dehydrogenase levels served as the control for equal protein loading.

SKP2, a well-characterized F-box protein, acts as a classic oncogene by promoting proliferation and survival of cancer cells, mainly through targeted degradation of numerous tumor suppressive proteins, including p21^33^ and p27^34,35^. We determined whether the increases of p21 and p27 protein levels were related to SKP2 changes caused by nobiletin. The results showed that SKP2 levels were significantly decreased in 786-O and 769-P cell lines by nobiletin treatment in a dose-dependent (**Figure 4A**) and time-dependent manner (**Figure 4B**). Next, we determined whether nobiletin downregulated SKP2 in a transcriptional and/or post-translational manner. To assess the effects of post-translational regulation, 786-O and 769-P cell lines were treated with nobiletin without or with co-treatment with a protease inhibitor MG-132, followed by SKP2 IB. In both RCC cell lines, MG-132 co-treatment did not rescue SKP2 expression to levels comparable to drug-free controls (**Figure 4C**). To further confirm the SKP2 degradation caused by nobiletin, we conducted cycloheximide chase analyses, showing that cycloheximide only slightly changed the rate of degradation of SKP2 protein in nobiletin-treated RCC cell lines (**Figure 4D**). However, we found that SKP2 mRNA levels were significantly reduced by nobiletin treatment of 786-O and 769-P cell lines (**Figure 4E**, *P* < 0.001). These results confirmed that nobiletin-induced SKP2 protein downregulation was mainly achieved by decreasing its transcriptional level. We also found that the transcription factor, FOXO3A, which is a negative regulator of the SKP2 and SKP2 SCF complex^36^, was gradually upregulated by escalating doses of nobiletin in 786-O and 769-P cell lines (**Figure 4F**). Based on these results, we speculated that the downregulation of SKP2 may be associated with increasing FOXO3A levels.

### The insensitivity of the CDK4/6 inhibitor, palbociclib, is associated with higher SKP2 levels in RCC cells

**Figure 5:**
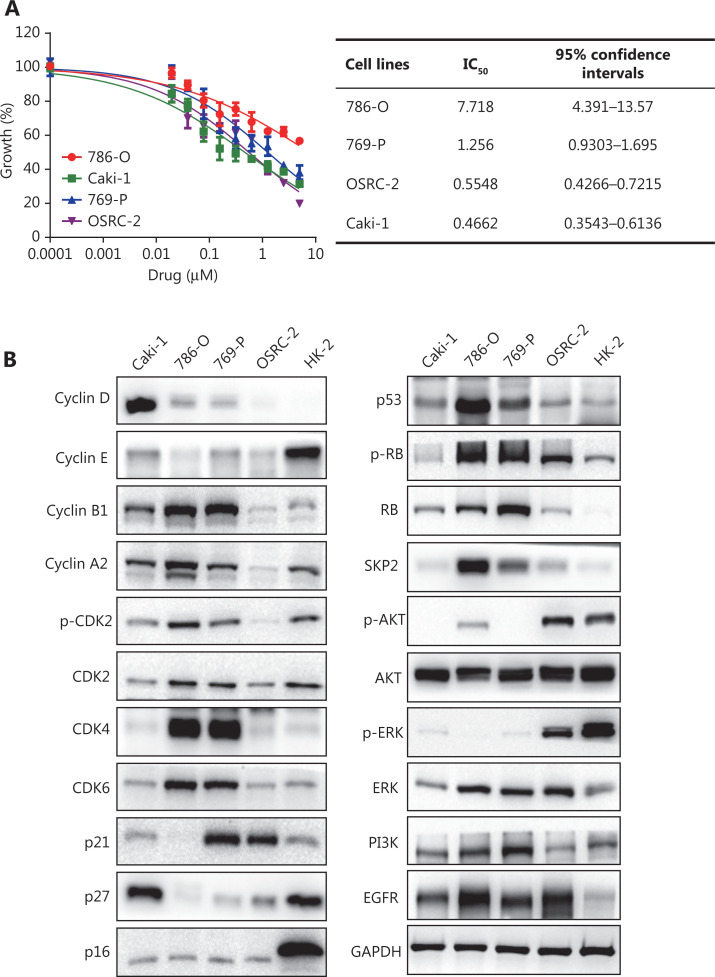
The anti-proliferative action of palbociclib on renal carcinoma cell (RCC) cell lines. (A) Dose-response curves of palbociclib treatment of RCC cell lines, after treatment for 48 h. Cell viability was detected using the Cell Counting Kit-8 (CCK-8) assay. (B) Basal levels of CDK2, CDK4, CDK6, SKP2 and other proteins in the RCC cell lines. RCC cell lines were cultured and followed by determination of protein expressions using IB analyses. Data are presented as the means ± SD. Glyceraldehyde 3-phosphate dehydrogenase levels served as the control for equal protein loading.

**Figure 6:**
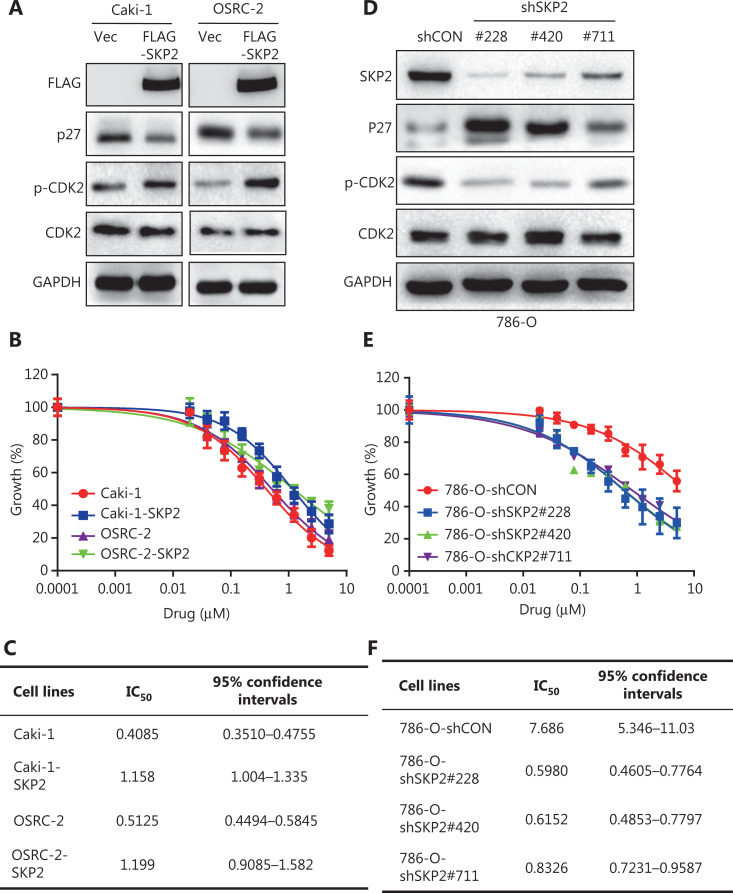
Sensitivity of palbociclib was associated with SKP2 levels in renal carcinoma cells (RCCs). (A) Overexpression of SKP2 attenuated p27 protein levels. The p27, p-CDK2, and CDK2 protein expressions were determined in stable clones of Caki-1 and OSRC-2 cell lines with ectopic expression of FLAG-tagged SKP2. (B–C) Overexpression of SKP2 decreased the sensitivity of palbociclib-treated RCC cells. Dose-response curves of palbociclib treatment of FLAG-tagged SKP2 stable clones and their corresponding vector controls after treatment for 48 h. The cell viability was detected using a Cell Counting Kit-8 (CCK-8) assay. (D) SKP2 silencing increased p27 protein levels. The 786-O cell line was transfected with either control siRNA, or shRNA targeting SKP2 (#228, #420, #711), then followed by determination of p27, SKP2, p-CDK2, and CDK2 expression using IB analyses. (E–F) SKP2 silencing increased the sensitivity of palbociclib-treated RCC cells. The 786-O cell line was transfected with either control siRNA, or siRNA targeting SKP2 (#228, #420, #711), and treated with different concentrations of palbociclib for 48 h. The cell viability was detected using CCK-8 assays. Data are presented as the means ± SD. Glyceraldehyde 3-phosphate dehydrogenase levels served as the control for equal protein loading.

Palbociclib, a CDK4/6-specific inhibitor, has shown clinical efficacy, but primary or secondary resistance has emerged as a problem^28^. Our results showed that the dose of palbociclib required to suppress 50% (IC_50_) of cell proliferation at 48 h was 0.4662 μM, 0.5548 μM, 1.256 μM, and 7.718 μM for the Caki-1, OSRC-2, 769-P, and 786-O cell lines, respectively (**Figure 5A**). To determine the reasons for the higher concentration of IC_50_ reactions in 786-O and 769-P cell lines, we detected the expressions of related proteins in 5 renal cell lines, namely the Caki-1, 786-O, 769-P, OSRC-2 and HK-2 cell lines. Higher levels of CDK4, CDK6, CDK2, p-CDK2, and SKP2, and lower levels of p27 were found in 786-O and 769-P cell lines (**Figure 5B**). The p27, as a tumor suppressor protein, can affect CDK2 activity^28^ and is directly degraded by SKP2^34,35^. The inhibition of RB phosphorylation caused by the CDK4/6 inhibitor, palbociclib, can be reversed by CDK2, which results in drug resistance. We therefore speculated that RCC cells with higher levels of SKP2 might require higher concentrations of palbociclib to achieve a IC_50_ response. We first overexpressed SKP2 in the Caki-1 and OSRC-2 cell lines, and then treated them with palbociclib for 48 h. The results showed that SKP2 overexpressed Caki-1 and OSRC-2 required higher concentrations of palbociclib to achieve an IC_50_ response, when compared with the control group (**Figure 6B and 6C**). The 786-O cells, a palbociclib highly-responding RCC cell line, was transfected with either control siRNA, or siRNA targeting SKP2 (#228, #420, or #711), followed by treatment with palbociclib for 48 h. The dose of palbociclib required to suppress 50% (IC_50_) of cell proliferation was 7.718 μM for the control group, which was at least 9-fold more than the SKP2 silencing group [IC_50_ (shSKP2-228) = 0.5980 μM; IC_50_ (shSKP2-420) = 0.6152 μM; IC_50_ (shSKP2-711) = 0.8326 μM] (**Figure 6D, 6E, and 6F**). From the IB results, we found that SKP2 overexpression significantly increased p-CDK2 levels by decreasing p27 levels in the Caki-1 and OSRC2 cell lines (**Figure 6A**), and *vice versa* (**Figure 6D**). Thus, we confirmed that the insensitivity of CDK4/6 inhibitor, palbociclib, was associated with higher SKP2 levels in RCC cells.

### A combination of nobiletin and palbociclib shows synergistic lethality *in vitro*

**Figure 7:**
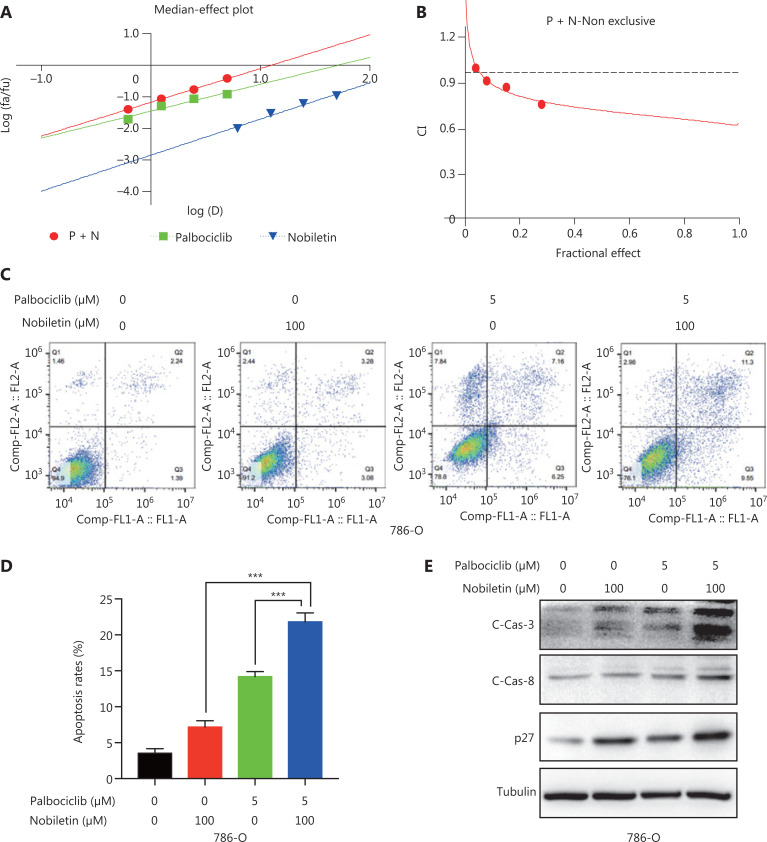
Nobiletin enhanced palbociclib-induced apoptosis in the 786-O cell line. (A–B) Nobiletin and palbociclib showed synergistic effects in the 786-O cell line. Combination index (CI)-effect plots and median effect plots were generated using CalcuSyn software. The points, a, b, c, and d, represent CI values for the combinations 6.25, 12.5, 25, and 50 μM nobiletin with 0.625, 1.25, 2.5, and 5 μM Palbociclb in a constant ratio, respectively. (C–D) The nobiletin-palbociclib combination strongly increased apoptosis in the 786-O cell line. Apoptosis in the 786-O cell line was examined after 48 h of treatment with dimethyl sulfoxide, 100 μM nobiletin, and/or 5 μM palbociclib by annexin V-fluorescein isothiocyanate/propidium binding, and analyzed by flow cytometry. (E) The nobiletin-palbociclib combination significantly increased apoptosis-related protein expression in the 786-O cell line. The 786-O cell line was examined after 48 h of treatment with dimethyl sulfoxide, 100 μM nobiletin, and/or 5 μM Palbociclb, and the expressions of cleaved caspase and p27 were determined using IB analyses. Data are presented as the means ± SD. ***P < 0.001, compared with the control group; *n* = 3. Tubulin levels served as the control for equal protein loading.

Clinical trials are currently ongoing with the single agent palbociclib in several advanced solid tumors^37^. As we previously discussed, nobiletin inhibited tumor progression by regulating the SKP2-p21/p27-CDK2 axis, and the insensitivity of the CDK4/6 inhibitor, palbociclib, was associated with higher SKP2 levels in RCC cells. Thus, we speculated that a combination of nobiletin and palbociclib may have the effects of dual inhibition of RCC cells. We selected 786-O and 769-P cell lines for the following experiments. Both CalcuSyn software and Jin’s formula were used as previously described to determine the synergy of the 2 agents. The 786-O was cultured with combinations of the 2 drugs at different doses but at a constant ratio (nobiletin to palbociclib: 6.25–0.625 μM, 12.5–1.25 μM, 25–2.5 μM, and 50–5.0 μM) for 48 h. The combination of 6.25 μM nobiletin with 0.625 μM palbociclib in the 786-O cell line inhibited cell proliferation by 32.0%, compared with monotherapy of nobiletin by 15.1% or palbociclib by 11.2%, indicating synergism (CI = 0.905; Q = 0.99; **Figure 7A**). Escalating doses, i.e., co-treatment with 12.5 μM nobiletin and 1.25 μM palbociclib (CI = 0.642; Q = 1.10) or 25 μM nobiletin and 2.5 μM palbociclib (CI = 0.585; Q = 1.19) or 50 μM nobiletin and 5.0 μM palbociclib (CI = 0.497; Q = 1.27), showed synergetic effects in 786-O cells (**Figure 7B**). Those findings showed that nobiletin and palbociclib had synergistic lethality, because they inhibited both CDK2 and CDK4/6 kinases. Furthermore, a combination of the 2 agents strongly induced apoptosis, when compared with a single agent in the 786-O and 769-P cell lines (nobiletin or palbociclib *vs.* nobiletin + palbociclib: *P* < 0.01; **Figure 7C and 7D and Supplementary Figure 3A and 3B**), which was further demonstrated by the increase of cleavage of caspase-8 and caspase-3 (**Figure 7E and Supplementary Figure S3C**). Similarly, a significant increase of p27 was observed with the combination treatment when compared with single agent (**Figure 7E and Supplementary Figure S3C**), suggesting that simultaneously inhibiting CDK2 and CDK4/6 kinases did more to induce apoptosis.

### A combination of nobiletin and palbociclib shows synergistic lethality *in vivo*

**Figure 8:**
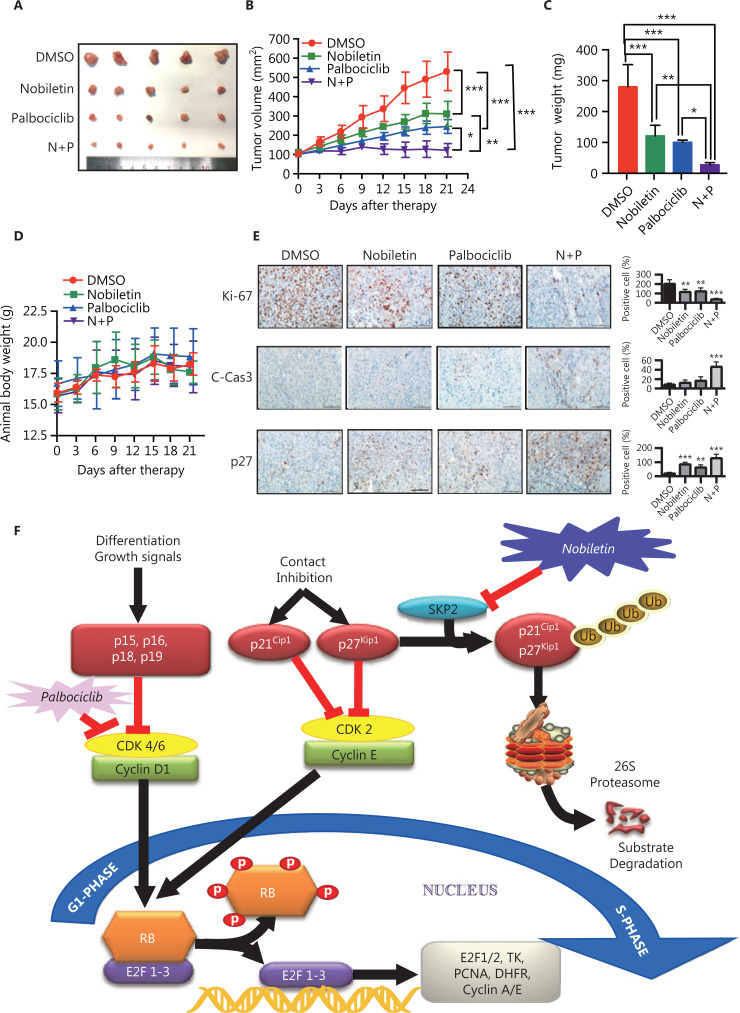
Nobiletin and palbociclib synergistically inhibited renal carcinoma cell (RCC) tumor growth in a xenograft model. (A–D) Synergistic antitumor activity of nobiletin and palbociclib in the 786-O xenograft model. The 786-O cell line was injected subcutaneously into the right flank side of nude mice. The mice were randomized when the tumor size reached about 100 mm^3^ and were treated as follows: vehicle, *n* = 5; nobiletin (40 mg/kg/days for every day for 3 weeks), *n* = 5; palbociclib (120 mg/kg/days for every day for 3 weeks), *n* = 5; nobiletin + palbociclib, *n* = 5. The tumor growth was monitored and the growth curve was plotted (B) and tumors were harvested and photographed (C). Body weight was measured during the treatment and plotted (D). (E) Immunohistochemical staining of xenograft tumor tissues. Tumor tissues from 4 groups of mice were fixed, sectioned, and stained with indicated antibodies. Scale bars: 100 μm. Data are presented as the means ± SD. *P < 0.05; **P < 0.01; ***P < 0.001, compared with the control group; *n* = 5. (F) Working model depicting the mechanisms of action underlying nobiletin induced anti-proliferation. Nobiletin-targeted SKP2 for transcription-mediated degradation to downregulate SKP2 levels, leading to the accumulation of tumor suppressor factors such as p21 and p27, which further inhibited the activity of G1 phase-related protein CDK2 leading to inhibition of cell proliferation in RCC cell lines. The combination of nobiletin and palbociclib showed a synergistic lethality by inhibiting the SKP2-p21/p27-CKD2-cyclin E-RB and CDK4/6-cyclin D-RB axis, respectively.

We validated the above *in vitro* findings by using an *in vivo* xenograft model. A 786-O xenograft model was established and treated with vehicle, nobiletin, and/or palbociclib. Consistent with the *in vitro* results, the combination of nobiletin and palbociclib suppressed tumor growth significantly more than single agent treatment (nobiletin or palbociclib *vs.* nobiletin + palbociclib: *P < 0.05; **P < 0.01; ***P < 0.001; **Figure 8A, 8B, and 8C**). The average tumor size and tumor weight at the end of the experiment (treatment for 21 days) were significantly lower in the nobiletin-palbociclib combination group (**Figure 8A and 8C**). The body weight of the xenograft model was unchanged during drug treatment, suggesting that the effect on normal tissues was minimal (**Figure 8D**). Finally, immunohistochemical staining of tumor tissues revealed that compared with nobiletin or palbociclib single agent treatment, a combination of the two agents inhibited cell growth (decrease of Ki-67 and increase of p27) and induced apoptosis (increase of cleaved caspase-3) (nobiletin or palbociclib *vs.* nobiletin + palbociclib: *P < 0.05; **P < 0.01; ***P < 0.001; **Figure 8E**) significantly more.

## Discussion

Polymethoxylated flavonoids (PMFs) have a variety of biological activities such as anti-cancer, anti-cardiovascular disease, prevention and treatment of obesity, and anti-inflammatory activities^38^, making them potential antitumor drugs^7^. Nobiletin is a common PMF that has been reported to prevent various tumor inductions and progressions^39,40^. Several studies have reported the antitumor activities of nobiletin, including inhibition of proliferation, induction of the cell cycle arrest, and promotion of apoptosis^11,41,42^.

In the present study, we found that nobiletin inhibited tumor proliferation and progression in RCC by regulating the SKP2-p21/p27-CDK2 axis. Nobiletin inhibited the proliferation of RCC lines in dose- and time-dependent manners (**Figure 1**) by inducing G1-S phase arrest in RCC cells (**Figure 2A and Supplementary Figure S1A**). The causal role of nobiletin in G1/S arrest was due to an increase of p27 and p21 and inhibition of CDK2 phosphatase, ultimately leading to a decrease of p-RB (**Figure 3A**). The p21 and p27 are crucial for restraining the G1-S phase transition^29,43^, and are regulated by the E3 ubiquitin ligase, SKP2^33–35^. Nobiletin caused accumulation of p27 and p21 levels by downregulating SKP2 protein expression in time- and dose-dependent manners by decreasing its transcriptional level (**Figure 4**), which might be due to nobiletin-induced upregulation of FOXO3A. FOXO3A is a transcriptional repressor of SKP2 gene expression, which directly binds to the SKP2 promoter^36^. Small molecules targeting SKP2 activity or SKP2 complex assembly have been examined in leukemia cells and in xenograft tumor models, respectively, and have been proven to be effective as anticancer agents^44,45^. These findings not only provide new insights into the molecular understanding of nobiletin’s anti-proliferative effects, but also highlight the potential of nobiletin in the development of SKP2-targeted anticancer therapeutics.

*De novo* or acquired resistance to CDK4/6 inhibitors is a common occurrence in anti-cancer treatment. Although palbociclib inhibited the proliferative activity in RCC cell lines, a few of the RCC cells were insensitive to palbociclib^23^. We found that palbociclib monotherapy inhibited cell proliferation significantly in Caki-1 (IC_50_ = 0.4662 μM) and OSRC-2 (IC_50_ = 0.5548 μM), but showed less effects in 769-P (IC_50_ = 1.256 μM) and 786-O (IC_50_ = 7.718 μM) cells, especially the 786-O cell line (**Figure 5A**). The insensitivities to 786-O and 769-P may be explained by the higher levels of p-CDK2 and SKP2 but lower levels of p27 (**Figure 5B**). CDK2/cyclinE could be deactivated by p27^28^, which could be targeted for degradation by SKP2^28,34,35^. A previous study confirmed that while estrogen receptor- (ER) positive breast cancer cell lines were inhibited by palbociclib, they quickly adapted because of degradation of p27 and a subsequent activation of CDK2, allowing compensatory phosphorylation of RB and passage into the S phase^28^. Another study also confirmed that insensitivity to CDK4/6 blockade was mediated by p-RB phosphorylation recovery induced by the noncanonical CDK2/cyclinD1 complex^27^. The cyclinE rebound is likely a consequence of CDK2/cyclinD1 activity and eventually triggering S-phase entry^27^. Based on these observations, searching for agents that reverse resistance of palbociclib represents a promising strategy to discover novel cancer therapeutics^28,46^. Herein, we showed that nobiletin downregulated SKP2 levels to suppress activation of CDK2 phosphatase (**Figure 3 and Figure 4**). A combination of nobiletin with palbociclib further inhibited cell proliferation and induced cell apoptosis by increasing p27 and proapoptotic protein levels, when compared to palbociclib treatment alone (**Figure 7E**). Similarly, a combination of nobiletin and palbociclib significantly inhibited tumor growth in a xenograft model (**Figure 8A, 8B, 8C, 8D, and 8E**).

It is noteworthy that natural compounds improved the sensitivity and efficacy of chemotherapy drugs^47–49^. It is also noteworthy that some natural compounds have been widely used in clinics, such as paclitaxel^50^. Nobiletin, as a natural extract, can be considered as a promising candidate for cancer therapy due to its multiple-targeting capabilities^11,51^. But the underlying mechanisms and bioavailability of nobiletin are still complex problems to understand^11,51,52^, which limits its application as a therapeutic agent. Our study demonstrated that nobiletin inhibited tumor progression by regulating the SKP2-p21/p27-CDK2-RB axis (working model, **Figure 8F**): SKP2 targets p27 for degradation, CDK2 can be reactivated upon p27 degradation, and phosphorylation of RB is recovered by activated CDK2, which leads to G1/S transition, tumor growth, and progression. However, nobiletin decreased SKP2 protein levels by reducing its transcriptional levels, and showed synergistic chemopreventive effects with palbociclib in RCC cells. The fact that a combination of nobiletin and palbociclib at their half doses produced stronger anti-cancer effects than nobiletin or palbociclib alone, provided the strong basis to utilize the nobiletin/palbociclib combination for renal cell carcinoma chemoprevention.

## Conclusions

Nobiletin downregulated the SKP2-p21/p27-CDK2 axis to inhibit tumor growth and progression in RCC cells. A combination of nobiletin with a CDK4/6 inhibitor may be a new therapeutic strategy to overcome the resistance of a single agent CDK4/6 inhibitor in cancer (especially RCC) treatments.
