# Supplementary material for: Nobiletin downregulates the SKP2-p21/p27-CDK2 axis to inhibit tumor progression and shows synergistic effects with palbociclib on renal cell carcinoma
Source: Cancer Biol Med. 2021 Feb 15;18(1):227–44. doi: 10.20892/j.issn.2095-3941.2020.0186 (PMC7877181; doi:10.20892/j.issn.2095-3941.2020.0186)
Supplement: Supplementary file 1 [file cbm-18-227-s001.pdf]

Supplementary materials

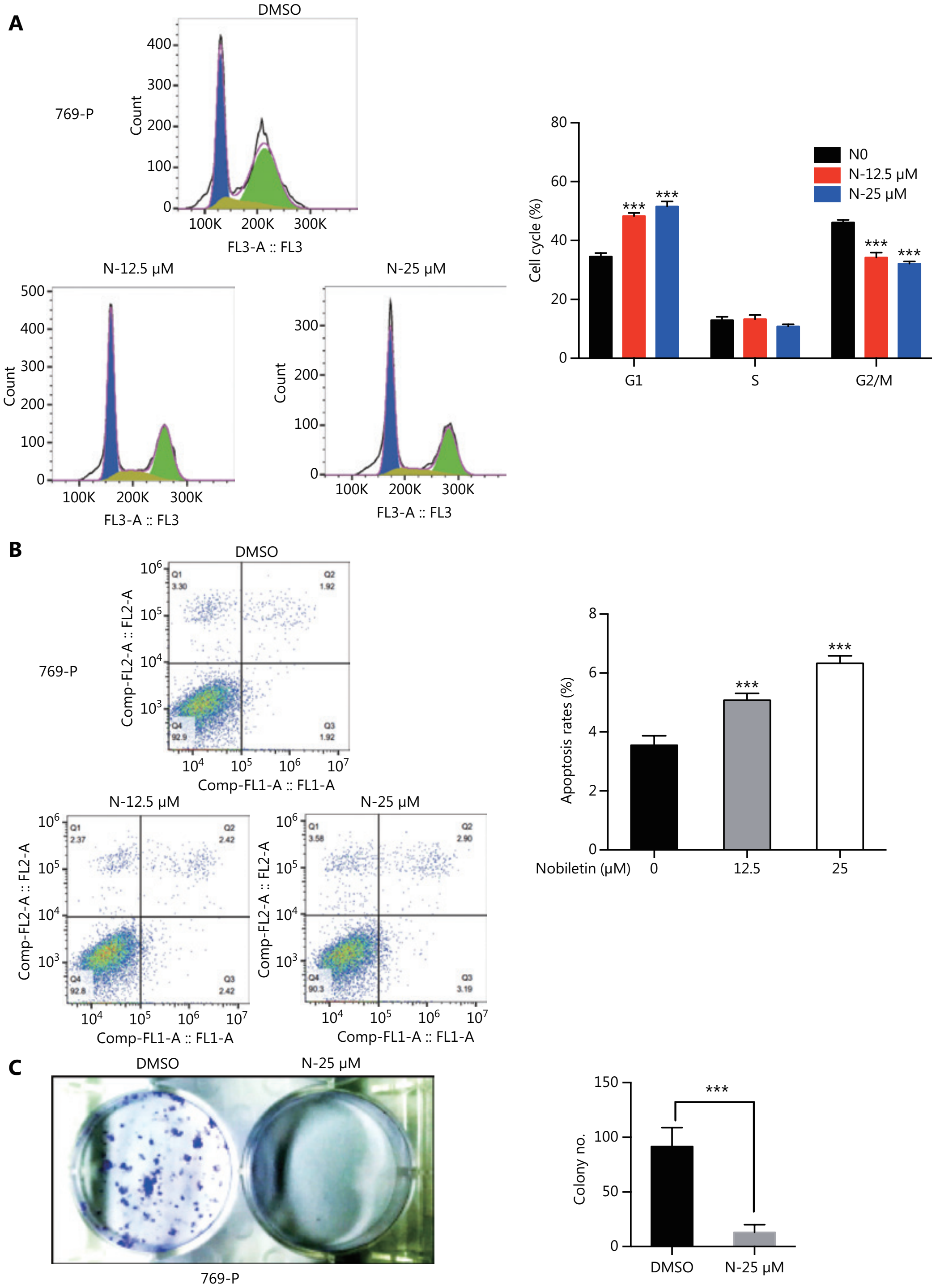

Figure S1 Nobiletin induced G1-phase cell cycle arrest, apoptosis, and colony-forming inhibition in 769-P cell line. (A) Nobiletin arrested cell cycle progression at the G1-phase cell cycle in 769-P cell line. 769-P cell line was treated with 12.5 or 25 μM nobiletin for 48 h, and cell cycle distributions were then analyzed by flow cytometry. (B) Nobiletin induced apoptosis in 769-P cell line. Apoptosis in 769-P cell line were examined after 48 h of treatment with 12.5 or 25 μM nobiletin by annexin V-FITC/PI binding and analyzed by flow cytometry. (C) Nobiletin

significantly suppressed colony formation in 769-P cell line. 769-P cell line treated with 25 μM nobiletin for 48 h were allowed to proliferate in drug-free culture media for 10~14 days to form colonies, followed by crystal violet staining for scoring colonies. Quantitative results were obtained from the number of colonies. Data were presented as means ± SD. ***P < 0.001, compared with the control group; n = 3.

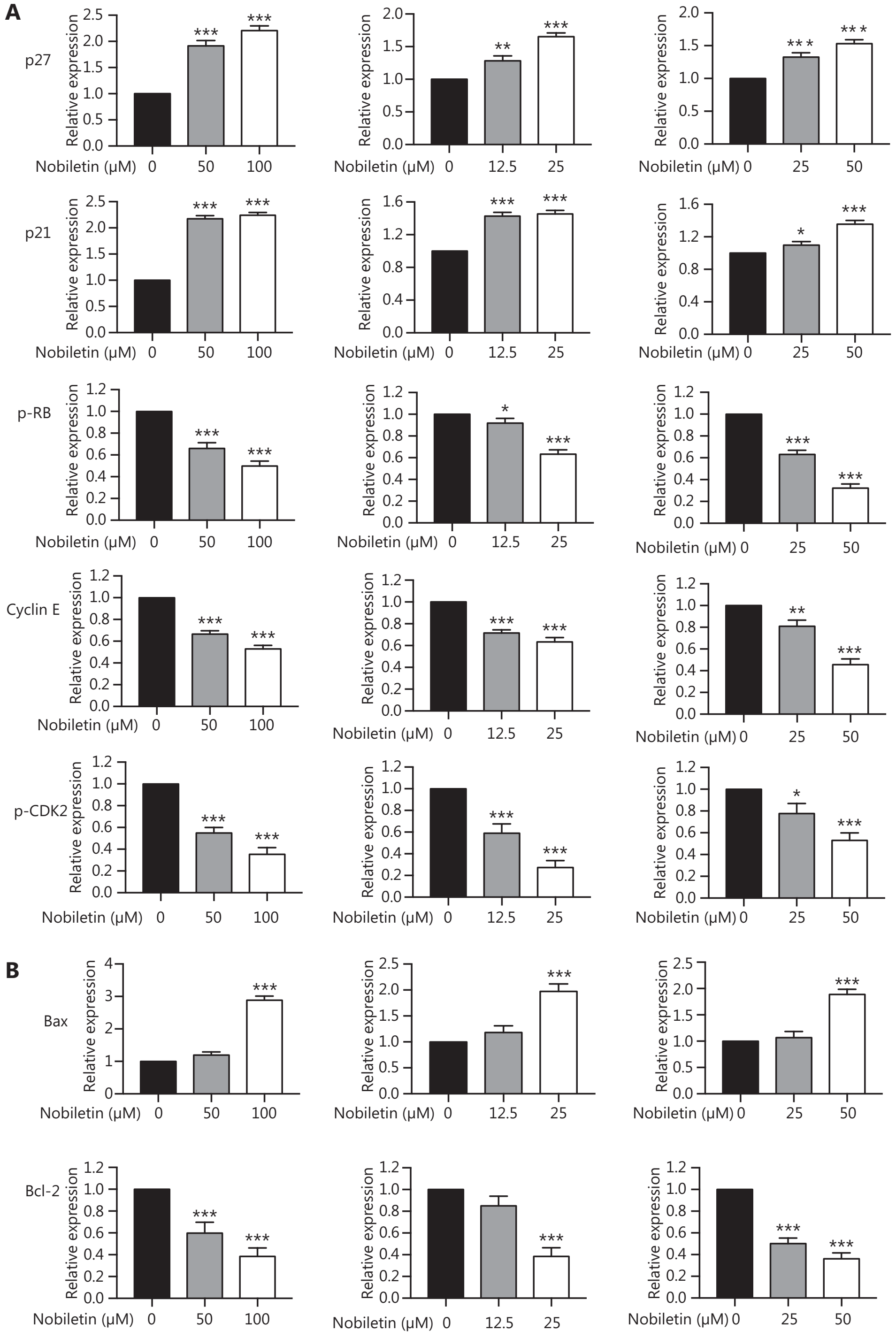

Figure S2 Effect of nobiletin on the cell cycle regulatory protein and apoptosis-related protein expression in RCC cell lines. (A) Effect of nobiletin on the cell cycle regulatory protein expression in RCC cell lines. RCC cell lines were treated with different doses of nobiletin (50 and

100 μM for 786-O cell line; 12.5 and 25 μM for 769-P cell line; 25 and 50 μM for OSRC-2 cell line) for 48 h, followed by determination of related-protein expression using IB analysis. (B) Effect of nobiletin on expression of apoptosis-related protein expression in RCC cell lines. RCC cell lines were treated with different doses of nobiletin (50 and 100 μM for 786-O cell line; 12.5 and 25 μM for 769-P cell line; 25 and 50 μM for OSRC-2 cell line) for 48 h, followed by determination of related-protein expression using IB analysis. Data were presented as means ± SD. *P < 0.05, **P < 0.01, ***P < 0.001, compared with the control group; n = 3. GAPDH levels served as the control for equal loading.

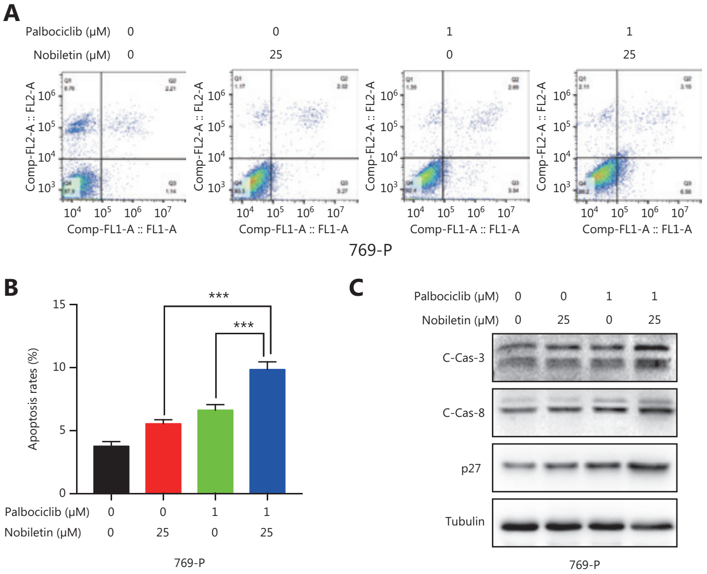

Figure S3 Nobiletin enhanced palbociclib-induced apoptosis in 769-P cell line. (A–B) Nobiletin-palbociclib combination strongly increased apoptosis in 769-P cell line. Apoptosis in 769-P cell line was examined after 48 h of treatment with DMSO, 25 μM nobiletin, and/or 1 μM palbociclib by annexin V-FITC/PI binding and analyzed by flow cytometry. (C) Nobiletin-palbociclib combination significantly increased apoptosis-related protein expression in 769-P cell line. 769-P cell line was examined after 48 h of treatment with DMSO, 25 μM nobiletin, and/or 1 μM palbociclib, and determined the expression of cleavages of caspase and p27 by IB analysis. Data were presented as means ± SD. ***P < 0.001, compared with the control group; n = 3. GAPDH levels served as the control for equal loading.
